# HSP90 Inhibitor 17-AAG Attenuates Nucleus Pulposus Inflammation and Catabolism Induced by M1-Polarized Macrophages

**DOI:** 10.3389/fcell.2021.796974

**Published:** 2022-01-04

**Authors:** Shuo Zhang, Peng Wang, Binwu Hu, Weijian Liu, Xiao Lv, Songfeng Chen, Zengwu Shao

**Affiliations:** ^1^ Department of Orthopaedics, Union Hospital, Tongji Medical College, Huazhong University of Science and Technology, Wuhan, China; ^2^ Department of Orthopaedics, The First Affiliated Hospital of Zhengzhou University, Zhengzhou, China

**Keywords:** intervertebral disc degeneration, nucleus pulposus cell, macrophage, inflammation, heat shock protein 90, 17-AAG

## Abstract

Overactivated inflammation and catabolism induced by proinflammatory macrophages are involved in the pathological processes of intervertebral disc (IVD) degeneration (IVDD). Our previous study suggested the protective role of inhibiting heat shock protein 90 (HSP90) in IVDD, while the underlying mechanisms need advanced research. The current study investigated the effects of HSP90 inhibitor 17-AAG on nucleus pulposus (NP) inflammation and catabolism induced by M1-polarized macrophages. Immunohistochemical staining of degenerated human IVD samples showed massive infiltration of macrophages, especially M1 phenotype, as well as elevated levels of interleukin (IL)-1β, tumor necrosis factor (TNF)-α and matrix metalloproteinase (MMP)13. The conditioned medium (CM) of inflamed NP cells (NPCs) enhanced M1 polarization of macrophages, while the CM of M1 macrophages but not M2 macrophages promoted the expression of inflammatory factors and matrix proteases in NPCs. Additionally, we found that 17-AAG could represent anti-inflammatory and anti-catabolic effects by modulating both macrophages and NPCs. On the one hand, 17-AAG attenuated the pro-inflammatory activity of M1 macrophages via inhibiting nuclear factor-κB (NF-κB) pathway and mitogen-activated protein kinase (MAPK) pathways. On the other hand, 17-AAG dampened M1-CM-induced inflammation and catabolism in NPCs by upregulating HSP70 and suppressing the Janus kinase 2 (JAK2)-signal transducer and activator of transcription 3 (STAT3) pathway. Moreover, both *in vitro* IVD culture models and murine disc puncture models supported that 17-AAG treatment decreased the levels of inflammatory factors and matrix proteases in IVD tissues. In conclusion, HSP90 inhibitor 17-AAG attenuates NP inflammation and catabolism induced by M1 macrophages, suggesting 17-AAG as a promising candidate for IVDD treatment.

## Introduction

Intervertebral disc (IVD) degeneration (IVDD) is a chronic, progressive disorder highly associated with low back pain (LBP). The microenvironment of degenerated IVD is featured with chronic inflammation, which contributes heavily to IVDD progression and LBP development (Risbud et al., 2014). Numerous studies supported the positive association between the levels of pro-inflammatory cytokines (such as interleukin (IL)-1β, tumor necrosis factor (TNF)-α, IL-6, IL-8, etc.) and the degree of IVDD (Lyu et al., 2021). Overactivated inflammation decreases the quantity and quality of IVD cells, and breaks the anabolic and catabolic balance of extracellular matrix (ECM) dynamics, resulting in structural and functional failures of IVD (Khan et al., 2017).

Macrophages, a class of immune effector cells present in multiple tissues, develop from circulating monocytes, and participate in inflammatory cascades (Murray et al., 2011). Under different stimuli, macrophages are polarized towards distinct phenotypes and exhibit distinct functions. Classically activated macrophages (M1 macrophages) work as powerful producers of multiple inflammatory cytokines, and mediate anti-infection and anti-tumor immunity. Alternatively activated macrophages (M2 macrophages) exert anti-inflammatory or immunoregulatory effects and enhance wound healing activities (Mosser and Edwards, 2008). The classical theory presumed that IVD is an immune-privileged organ, and circulating immune cells rarely influence the physiology and pathology of IVD. However, emerging evidence supported that macrophages could be recruited into IVD regions, especially in degenerated or herniated IVD tissues (Shamji et al., 2010).

Heat shock protein 90 (HSP90), a family of highly conserved molecular chaperones, modulates the biological activities of client proteins, thereby regulating various cellular processes, including growth, survival, differentiation and proteostasis (Schopf et al., 2017). Our previous study reported that HSP90 inhibition prevented compression-induced nucleus pulposus (NP) stem cells (NPSCs) death (Hu et al., 2020). Moreover, inhibiting HSP90 could impede cellular inflammation responses by not only activating anti-inflammatory effector HSP70 but also modulating client proteins degradation to suppress inflammatory signaling cascades (Tukaj et al., 2016). The chaperone function of HSP90 plays a vital role in the activation of Janus kinase 2 (JAK2)-signal transducer and activator of transcription 3 (STAT3) pathway, which is involved in the inflammation and catabolism of IVD cells (Marubayashi et al., 2010; Suzuki et al., 2017). Correspondingly, anti-HSP90 therapy emerged as a potential strategy to reverse the pro-inflammatory phenotype in multiple autoimmune/inflammatory diseases, such as rheumatoid arthritis (Rice et al., 2008), autoimmune dermatitis (Tukaj et al., 2017), etc.

Previous studies demonstrated that macrophages infiltrated into IVD tissues during IVDD progression and regulated the inflammatory microenvironment of IVD (Shamji et al., 2010; Nakazawa et al., 2018). However, the interactions between different phenotypes of macrophages and IVD cells have not been clearly elucidated, and targeted treatment strategies are insufficient to date. The present study aimed to explore the role of macrophages in NP inflammation and catabolism, and to address the anti-inflammatory and anti-catabolic effects of HSP90 inhibitor 17-AAG as well as underlying mechanisms.

## Materials and Methods

### Single-Cell RNA Sequencing Data Analysis

A copy of previously published single-cell RNA sequencing data (GSE154884) using the 10X Genomics technology was further analyzed to detect macrophage population in IVD tissues (Wang et al., 2020). The sequencing count matrix was derived from the IVD samples of four male and four female wild-type Sprague-Dawley rats (8-week-old, 450 g). The quality control process was performed using Seurat package (Version 3.0.1) (Butler et al., 2018). In brief, cells with less than 300 or more than 7,000 unique molecular identifiers (UMIs), or cells with more than 10% mitochondrion-derived UMIs were excluded. Finally, 11,764 single cells with 15,407 genes were subjected to the following analyses. The main cell clusters were identified using the unsupervised FindClusters function in Seurat package with resolution at 1.2, and visualized in 2D model using T-distributed Stochastic Neighbor Embedding (t-SNE) method (Aliverti et al., 2020). FindAllMarkers function was used for the annotation of these clusters, based on the CellMarker database (Zhang et al., 2019).

### Collection of Human IVD Tissues

Human IVD specimens were derived from twelve patients undergoing selective operations due to lumbar disc herniation in the Department of Orthopaedics, Wuhan Union Hospital. Written informed consents were obtained from all the relevant patients. IVD samples were fixed with 4% formaldehyde and embedded in paraffin for histological analysis. Based on preoperative magnetic resonance imaging (MRI), the IVD tissues were divided into two groups: mildly degenerated IVD (Grade I–II) and severely degenerated IVD (Grade III–V) according to Pfirrmann grade system (Pfirrmann et al., 2001). The characteristics of enrolled patients were listed in Supplementary Table S1.

### Culture and Polarization of RAW264.7 Macrophages

The murine macrophage cell line RAW264.7 (RAW) was cultured in DMEM-High Glucose (HyClone, Logan, UT, United States) with 10% fetal bovine serum (FBS; Gibco, CA, United States) and 1% penicillin/streptomycin at 37°C, in humidified atmosphere with 5% CO_2_. Untreated RAWs were defined as M0 macrophages. To induce M1 or M2 polarization, RAWs were treated with 500 ng/ml lipopolysaccharide (LPS) (Beyotime, Shanghai, China) or 30 ng/ml murine IL-4 (PeproTech, Rocky Hill, NJ, United States) for 24 h, respectively.

### Isolation and Culture of NP Cells (NPCs)

Mature female Sprague-Dawley rats (weighing 200–250 g) were obtained from the Experimental Animal Center of Tongji Medical College, Huazhong University of Science and Technology (Wuhan, China). The rats were euthanized by cervical dislocation after anesthesia (pentobarbital 30 mg/kg b.w., i.p.). Then, lumbar spines were harvested and each disc was cut transversely to separate gelatinous NP tissues. NP samples were washed with phosphate buffer solution (PBS) for three times, minced into small fragments, and then digested in 0.2% (m/v) type II collagenase (Sigma-Aldrich, St. Louis, MO, United States) for 15 min at 37°C. Subsequently, digested NP tissues were centrifuged at 1,400 rpm for 5 min, resuspended and cultured in DMEM/F-12 (HyClone, Logan, UT, United States) with 10% FBS and 1% penicillin/streptomycin at 37°C, in humidified atmosphere with 5% CO2. The culture medium was changed every 3 days. The cells were trypsinized with 0.25% trypsin-EDTA (Gibco) for subculture at 80–90% confluence. The second passage of NPCs was used in the following experiments.

### Collection of the Conditioned Medium of RAWs and NPCs

After the induction of M1 or M2 polarization, the medium of RAWs was changed to serum-free medium for an additional 24 h culture. Then, the CM was centrifuged at 2,000 g for 10 min at 4°C to remove cellular debris, and stored at -80°C for subsequent experiments. Repeated freeze-thaw cycles were not allowed, and the storage time was less than 72 h to avoid cytokine inactivation. The CM from different phenotypes of macrophage were defined as M0-CM, M1-CM and M2-CM, respectively. As for the collection of NPC CM, NPCs were stimulated with 20 ng/ml rat IL-1β (PeproTech) for 24 h and then the medium was changed. After another 24 h, the CM of untreated and IL-1β-treated NPCs were harvested and defined as control NPC-CM and IL-1β-treated NPC-CM, respectively.

### Treatments of RAWs and NPCs

M0 RAWs were cultured with control NPC-CM and IL-1β-treated NPC-CM for 36 h to explore the effects of NPC-derived factors on macrophage polarization. In another experiment, M1-polarized macrophages were treated with 200, 500 and 750 nM 17-AAG (Selleck, Houston, TX, United States) to evaluate the effects of 17-AAG on the M1 polarization of macrophages. NPCs were incubated for 36 h with macrophage CM. In M1-CM groups, NPCs were treated with 100, 200 and 500 nM 17-AAG, 1 μM STAT3 inhibitor Stattic (Selleck) and 1 μM HSP70 activator TRC051384 (TRC) (Selleck).

### Quantitative Real-Time PCR Analysis

After the designated treatments, RNA was extracted from NPCs or RAWs by the TRIzol reagent (Vazyme Biotech, Nanjing, China). Then, the concentrations of the total RNA were examined by the Nanodrop 2000. The isolated RNA was reverse-transcribed into complementary DNA (cDNA) by a Reverse Transcription Kit (Vazyme Biotech). The gene expression levels were quantified by a SYBR Prime Script RT-PCR Kit (Vazyme Biotech) on the Step One Plus Real-Time PCR system (Bio-Rad, Hercules, CA, United States). Relative quantities of target genes were normalized to *Gapdh* levels and calculated using the -ΔΔCt method, as previously reported (Schmittgen et al., 2008). A -ΔΔCt > 0 indicated that the expression of the target gene in the treatment group was more abundant than that in corresponding control group. The primer sequences used in the current study were listed in Supplementary Table S2.

### Western Blot Analysis

After treatments, NPCs or RAWs were harvested, and lysed by the radioimmunoprecipitation assay (RIPA) lysis buffer (Beyotime, Shanghai, China) with a cocktail of protease and phosphatase inhibitors. Protein concentration was determined by an Enhanced BCA Protein Assay Kit (Beyotime). Then, protein samples were electrophoresed in sodium dodecyl sulfate-polyacrylamide gel (SDS-PAGE) and transferred onto polyvinylidene difluoride (PVDF) membranes (EMD Millipore, Billerica, MA, United States). After being blocked in 5% (m/v) bovine serum albumin for 1 h at room temperature, the membranes were incubated with primary antibodies overnight at 4°C. Subsequently, the membranes were washed with Tris-buffered solution with Tween 20 (TBST) for three times and incubated with corresponding horseradish peroxidase-conjugated secondary antibodies for 1 h at room temperature. The expression levels of proteins were detected using Electro-Chemi-Luminescence (ECL) detection reagent (Affinity Biosciences, OH, United States) according to the manufacturer’s instructions. The integrated density of gray-value for each western blot (WB) band was analyzed by ImageJ software (Bethesda, MD, United States). The density ratios of target proteins and GAPDH (internal control) were calculated, and the relative expression quantities of control groups were normalized to 1. The antibodies used in the current study were listed in Supplementary Table S3.

### Co-Immunoprecipitation

Protein A/G Magnetic Beads (Bimake) were incubated with mouse anti-HSP90α/β antibody or control mouse IgG. Then, the beads were used to pull down the immunoprecipitates from cell lysates, and magnetized to discard the supernatant. After being thoroughly washed with lysis buffer, the beads were boiled in loading buffer and the pull-down proteins were revealed with antibodies of HSP90α, HSP90β, JAK2 and STAT3 by WB analysis.

### Immunofluorescence Staining

NPCs seeded on glass coverslips were washed with PBS, fixed in 4% paraformaldehyde for 15 min, permeabilized with 0.5% Triton X-100 (Beyotime) for 15 min, and blocked with goat serum for 1 h at room temperature. Subsequently, cells were incubated with rabbit anti-p-STAT3 antibody (1:300) or the mixture of mouse anti-matrix metalloproteinase (MMP)3 antibody (1:300) and rabbit anti-MMP13 antibody (1:300) at 4°C overnight, followed by the incubation with Cy3-conjugated goat anti-rabbit antibody (1:300; Servicebio, Wuhan, China) or the mixture of FITC-conjugated goat anti-mouse antibody (1:300; Servicebio) and Cy3-conjugated goat anti-rabbit antibody (1:300). Finally, the nuclei were counterstained with 4,6-diamidino-2-phenylindole (DAPI) before being detected by a fluorescence microscope (Olympus IX71, Tokyo, Japan).

### Immunohistochemical Staining

The paraffin-embedded samples were cut into 4 μm thick sections. The sections were deparaffinized, rehydrated and incubated with 3% H_2_O_2_ for 20 min at room temperature. Antigen retrieval was performed by microwaving in Tris-EDTA buffer (pH 9.0) for 15 min. Then, the sections were blocked with goat serum for 30 min at 37°C, followed by the incubation with anti-CD68 (1:100), anti-F4/80 (1:800), anti-CD86 (1:900), anti-CD206 (1:600), anti-IL-1β (1:250), anti-TNF-α (1:100), anti-MMP13 (1:250) antibodies at 4°C overnight. Non-immune rabbit IgG at the same dilution as the primary antibody was used as negative controls. After being washed, the sections were labelled with horseradish peroxidase-labeled goat anti-rabbit antibody at 37°C for 40 min, visualized with diaminobenzidine (DAB) and counterstained with hematoxylin.

### *In vitro* Organ Culture Model of Murine Lumbar IVD Tissues

*In vitro* organ culture of murine IVD was conducted as previously described (Liu et al., 2017). Twenty-three male C57BL/6J mice (3 months old) (three for macrophage-IVD co-culture, twenty for macrophage CM treatment) were euthanatized by cervical dislocation after anesthesia (pentobarbital 30 mg/kg b.w., i.p.), lumbar spines were harvested under sterile conditions, and cultured in DMEM/F-12 with 20% FBS and 1% penicillin/streptomycin at 37°C. The culture medium was changed every 2 days. Transwell culture plates with permeable membrane of 0.4 μm pore size, which allowed the exchange of soluble factors, were used to establish macrophage-IVD co-culture system. RAWs were seeded in the lower compartment, and lumbar IVD tissues were placed in the upper compartment. The co-culture treatment was maintained for 3 days and RAWs were harvested for further testing. In another experiment, murine lumbar IVDs were treated with macrophage CM. Twenty mice were randomly assigned into four groups (five mice per group): control group, M1-CM group, M2-CM group, and M1-CM/17-AAG (500 nM) group. L2/3, L3/4 and L4/5 vertebrae-disc-vertebrae functional spinal units of each mouse were separated using aseptic techniques. After 10 days *in vitro* culture, IVD samples were collected for histological detections.

### Puncture-Induced IVDD Model

Animal experiments were approved by the Medical Ethics Committee of Tongji Medical College, Huazhong University of Science and Technology. The twenty-four male C57BL/6J mice (3 months old) were randomly divided into three groups (8 mice each group), and anesthetized with pentobarbital (30 mg/kg b.w., i.p.). The mice in group 1 were subjected to sham operation, which was performed by puncturing through the skin without injuring IVDs. The coccygeal (Co) 6/7 and 7/8 IVDs of group 2 and group 3 were percutaneously punctured by a 30-gauge needle parallel to the endplates. Then, the needle was rotated 180° and remained in the disc for 30 s. As for drug administrations, mice in group 3 were injected intraperitoneally with 17-AAG (40 mg/kg b.w.) three times per week, every other day except the seventh day after the surgery, as previously reported (Siebelt et al., 2013). For group 1 and group 2, equivoluminal DMSO was injected intraperitoneally. After 8 weeks, Co6/7 and Co7/8 IVD samples were fixed in 4% formaldehyde, decalcified with EDTA decalcifying solution.

### Statistical Analysis

For *in vitro* experiments on NPCs, the NPCs in the three independent experiments were derived from different donors. As for RAW experiments, WB analysis and qRT-PCR were repeated three times by different researchers (Figure 2, Supplementary Figures S1,S2). All experimental data were expressed as the mean ± standard deviation (SD) and statistical analysis was conducted on GraphPad Prism 7.00. Student’s *t-*test (two groups) or one-way analysis of variance (ANOVA) followed by the least significant difference (LSD) test (three or more groups) were used to analyze the statistical difference. A *p*-value < 0.05 was considered statistically significant.

## Results

### Detections of Macrophages in IVD Tissues

**FIGURE 1:**
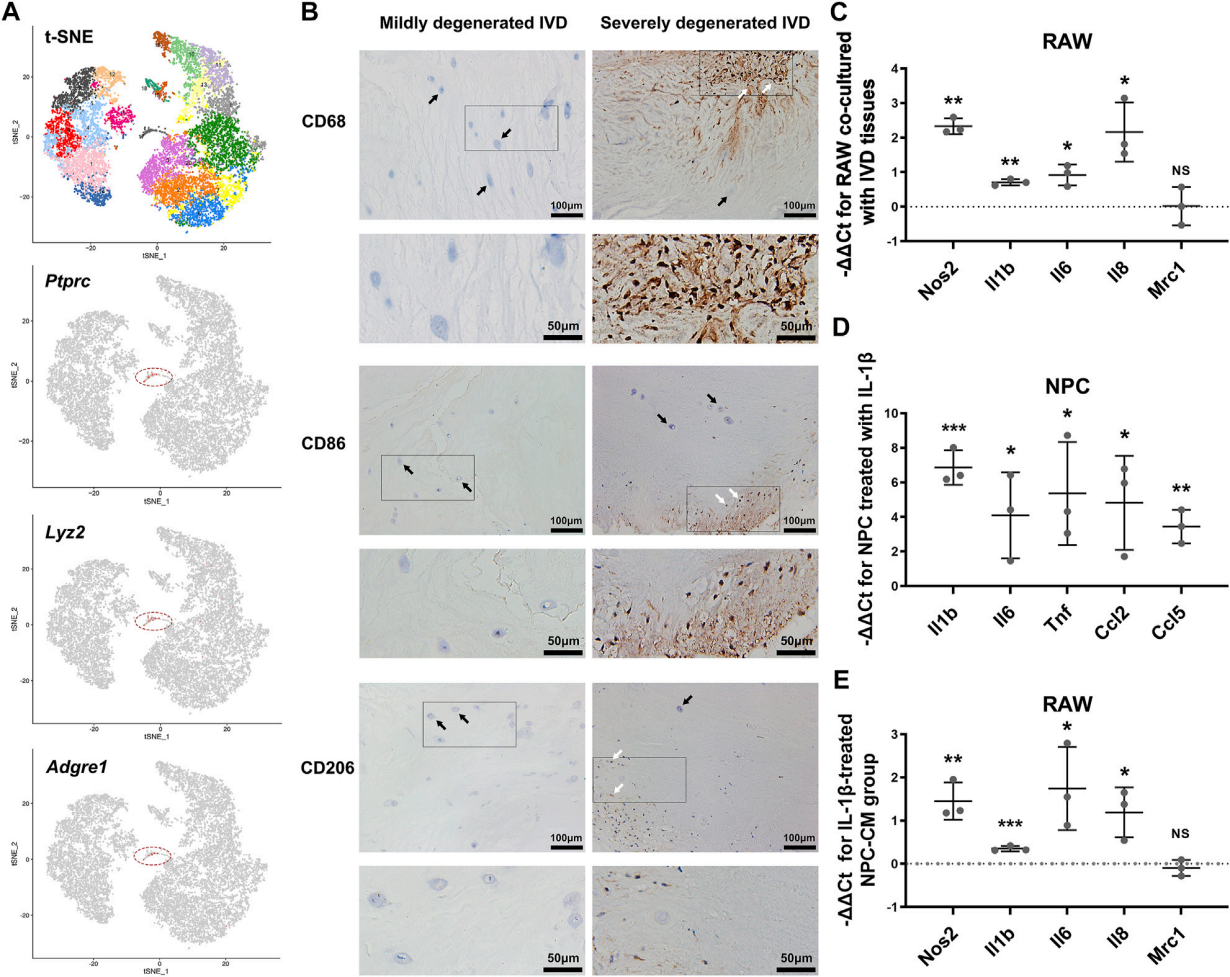
The infiltration and polarization of macrophages into IVD tissues. **(A)** The t-SNE figure of 11,764 single cells of rat IVD tissues, and the expression levels of *Ptprc*, *Lyz2* and *Adgre1* (gray: low expression, red: high expression, circled cell cluster: Cluster 17). **(B)** IHC staining for CD68, CD86 and CD206 in mildly degenerated and severely degenerated human IVD tissues (white arrowheads: immunostaining-positive cells, black arrowheads: IVD cells). **(C)** The relative mRNA levels of *Nos2*, *Il1b*, *Il6*, *Il8* and *Mrc1* in RAW co-cultured with IVD tissues vs M0 RAW. **(D)** The relative mRNA levels of *Il1b*, *Il6*, *Tnf*, *Ccl2* and *Ccl5* in NPC stimulated with IL-1β vs. control NPC. **(E)** The relative mRNA levels of *Nos2*, *Il1b*, *Il6*, *Il8* and *Mrc1* in RAW cultured with IL-1β-treated NPC-CM vs. RAW cultured with control NPC-CM. C-E, data were presented as Mean ± SD of three replicates. (*p < 0.05, **p < 0.01, ***p < 0.001. NS, not significant).

Previously published single-cell RNA sequencing data of rat IVDs were further analyzed (Wang et al., 2020). We identified a cluster of myeloid-like cells (Cluster 17) (1.33%), which specifically expressed the marker genes of immune cells (*Ptprc*, also known as *Cd45*), and myeloid cells (*Lyz2*). (Figure 1A) Additionally, macrophage marker *Adgre1* (also known as *Emr1*)-positive cells were also detected.

To further explore the association between IVDD degree and macrophage infiltration, we conducted immunohistochemical (IHC) staining for CD68, CD86 and CD206 in human lumbar IVD tissues. Compared to mildly degenerated IVD tissues, the severely degenerated discs showed massive infiltration of CD68-positive macrophages. As for the subtypes of macrophages, cells expressing CD86 (indicating M1 macrophages) and CD206 (indicating M2 macrophages) were identified only in severely degenerated discs. CD86-positive cells were more present than CD206-positive cells (Figure 1B).

### Effects of IVD Tissues and Inflamed NPCs on Macrophages Polarization

The phenotype shift of macrophages is highly related with the function states. Based on PCR results, LPS-induced M1 macrophages expressed higher levels of *Nos2*, *Il1b*, *Il6*, *Il8* and *Tnf* compared with M0 macrophages, while *Mrc1* and *Il4* were markedly elevated in IL-4-induced M2 macrophages. (Supplementary Figure S1).

We established a macrophage-IVD co-culture system to evaluate the modulation of macrophage phenotype by IVD tissues. In presence of IVD tissues, the levels of *Nos2*, *Il1b*, *Il6* and *Il8* were increased in RAWs to varying degrees, indicating an enhanced tendency toward M1 polarization. However, the results showed no obvious change in the expression of M2 macrophage marker *Mrc1* (Figure 1C).

Secreted factors from inflamed NP cells may serve as crucial regulators of macrophage polarization. PCR showed that IL-1β treatment activated the expression of *Il1b*, *Il6*, *Tnf*, *Ccl2* and *Ccl5* in NPCs. (Figure 1D) Moreover, RAWs represented a tendency to polarize to M1 phenotype with increased mRNA expression of *Nos2*, *Il1b*, *Il6* and *Il8* after stimulated by IL-1β-treated NPC-CM (Figure 1E).

### 17-AAG Inhibits M1 Polarization of Macrophages by Targeting MAPK and NF-κB Pathways

**FIGURE 2:**
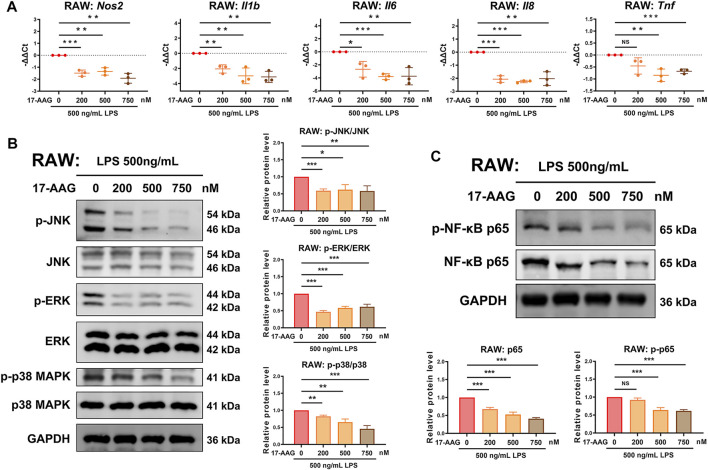
17-AAG dampens the M1 polarization of macrophages. **(A)** The mRNA levels of *Nos2*, *Il1b*, *Il6*, *Il8* and *Tnf* in RAWs. **(B,C)** Representative WB graphs and statistical analysis of p-JNK, JNK, p-ERK, ERK, p-p38 MAPK, p38 MAPK, p-NF-κB p65, NF-κB p65 and GAPDH in RAWs. Data were presented as Mean ± SD of three repeated experiments. (*p < 0.05, **p < 0.01, ***p < 0.001. NS, not significant).

To investigate the effects of 17-AAG on M1 polarization of macrophages, we conducted qRT-PCR analysis to measure the expression of M1 macrophage-related genes. The results showed that 17-AAG decreased the levels of *Nos2*, *Il1b*, *Il6*, *Il8* and *Tnf* in M1 RAWs. (Figure 2A) Previous study reported that the MAPK and NF-κB pathways were markedly activated in M1- but not M2-polarized macrophages, and were involved in the phenotype maintenance of M1 polarization (Zhou et al., 2019). WB analysis revealed that 17-AAG inhibited the phosphorylation of JNK, ERK and p38 MAPK in RAWs dose-dependently. (Figure 2B) In the NF-κB pathway, the levels of both NF-κB p65 and p-NF-κB p65 were downregulated by 17-AAG treatment. (Figure 2C) Furthermore, the expression levels of HSP90α and HSP90β in RAWs were not markedly changed after 200, 500 and 750 nM 17-AAG treatment. (Supplementary Figure S2) Taken together, these data indicated that 17-AAG impeded M1 polarization of macrophages by inhibiting MAPK and NF-κB pathways.

### 17-AAG Ameliorates the Pro-inflammatory Phenotype of NPCs Induced by M1-CM

**FIGURE 3:**
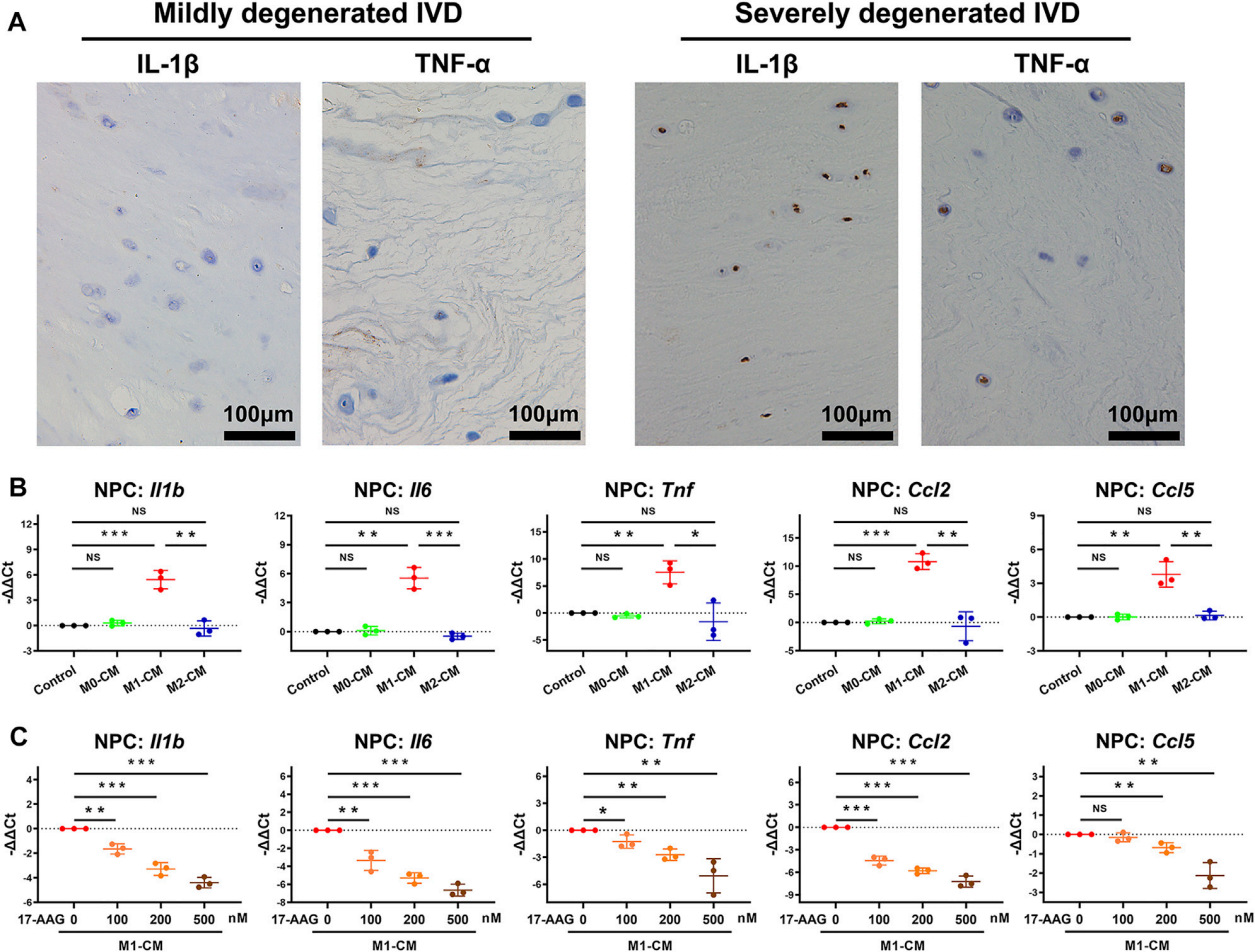
17-AAG suppresses the pro-inflammatory phenotype of NPCs induced by M1-CM. **(A)** IHC staining for IL-1β and TNF-α in mildly degenerated and severely degenerated human IVD tissues. **(B,C)** The mRNA levels of *Il1b*, *Il6*, *Tnf*, *Ccl2* and *Ccl5* in NPCs. **(B,C)**, data were presented as Mean ± SD of three replicates. (*p < 0.05, **p < 0.01, ***p < 0.001. NS, not significant).

We explored the effects of macrophages CM and 17-AAG on the inflammatory phenotype of NPCs. IHC staining showed that the expression levels of IL-1β and TNF-α increased in the IVD cells of severely degenerated human IVD tissues. (Figure 3A) Under the stimulation of M1-CM, NPCs express elevated levels of *Il1b*, *Il6*, *Tnf*, *Ccl2* and *Ccl5*, while treatment with 17-AAG partially reverse these changes. (Figures 3B,C)

### 17-AAG Ameliorates the Catabolic Phenotype of NPCs Induced by M1-CM

**FIGURE 4:**
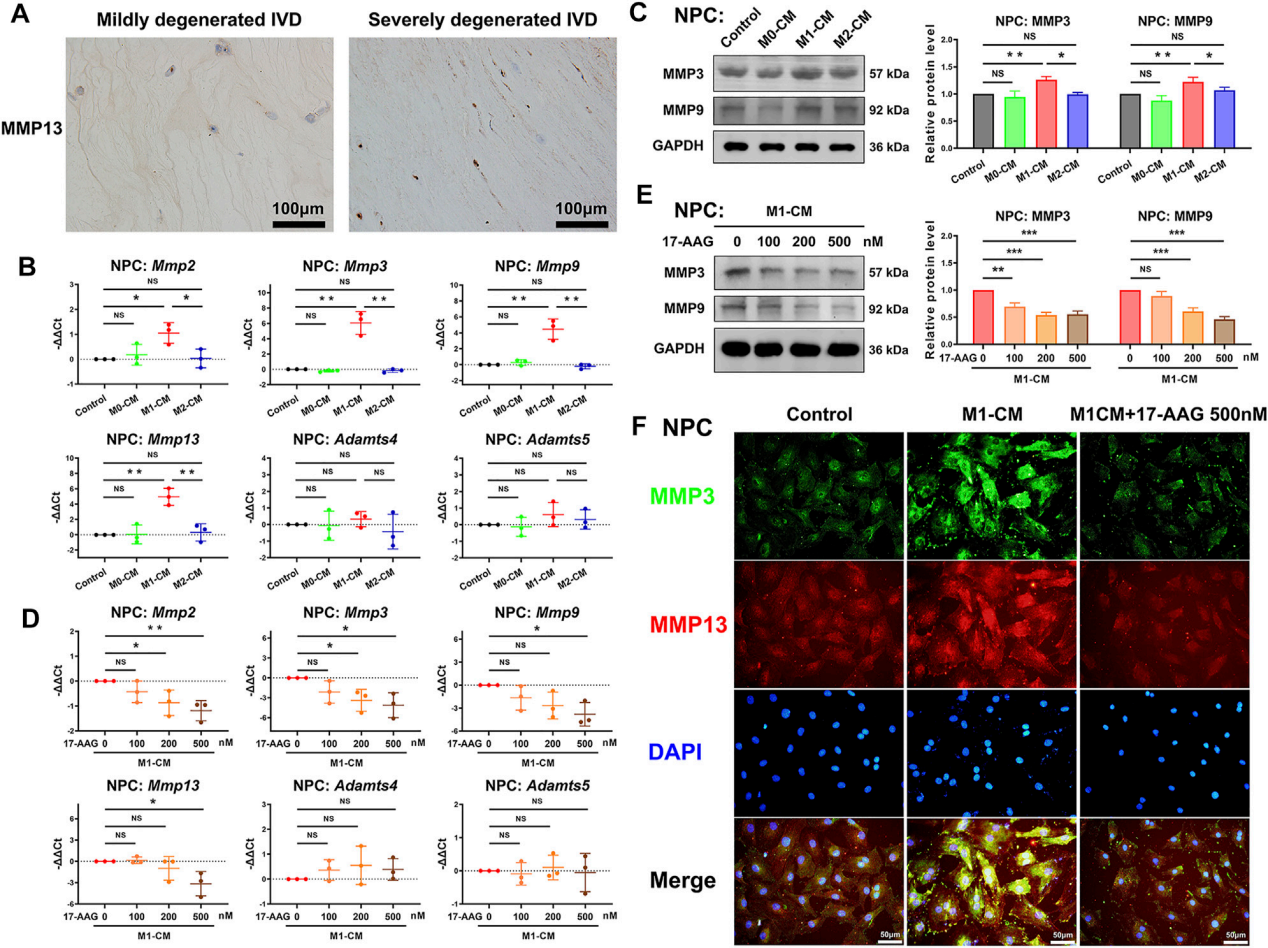
17-AAG attenuates the catabolic phenotype of NPCs induced by M1-CM. **(A)** IHC staining for MMP13 in mildly degenerated and severely degenerated human IVD tissues. **(B,D)** The mRNA levels of *Mmp2*, *Mmp3*, *Mmp9*, *Mmp13*, *Adamts4* and *Adamts5* in NPCs. **(C,E)** Representative WB graphs and statistical analysis of MMP3, MMP9 and GAPDH in NPCs. **(F)** Typical fluorescence photomicrographs of IF staining for MMP3 and MMP13 in NPCs. **(B–E)**, data were presented as Mean ± SD of three replicates. (*p < 0.05, **p < 0.01, ***p < 0.001. NS, not significant).

MMPs and a disintegrin and metalloproteinase with thrombospondin motifs (ADAMTSs) are two main families of catabolic proteases, which participate in the breakdown of ECM. IHC analysis of human discs detected that severely degenerated IVD tissues expressed higher level of MMP13 than mildly degenerated IVD tissues. (Figure 4A) As shown in Figure 4B, M1-CM treatment increased the mRNA levels of *Mmp2*, *Mmp3*, *Mmp9* and *Mmp13*, but not *Adamts4* and *Adamts5* in NPCs compared with control group. WB analysis further revealed that M1-CM promoted the expression of MMP3 and MMP9 in NPCs. (Figure 4C) When M1-CM-stimulated NPCs were treated with 17-AAG, the mRNA levels of *Mmp2*, *Mmp3*, *Mmp9* and *Mmp13* were markedly decreased. (Figure 4D) Moreover, WB analysis (Figure 4E) and immunofluorescence (IF) staining (Figure 4F) also supported the inhibiting effects of 17-AAG on the expression of MMPs in NPCs.

Collagen II is one of the main ECM components of NP tissues. Based on the results of PCR, M1-CM but not M2-CM decreased the levels of *Col2a1* in NPCs, and 17-AAG prevented the decline of *Col2a1* induced by M1-CM. (Supplementary Figures S3A,B) These results were also confirmed by WB analysis. (Supplementary Figures S3C,D) Taken together, 17-AAG is proved to be a promising agent to protect the anabolic and catabolic balance of ECM dynamics.

### 17-AAG Upregulates the Expression of HSP70 in NPCs

**FIGURE 5:**
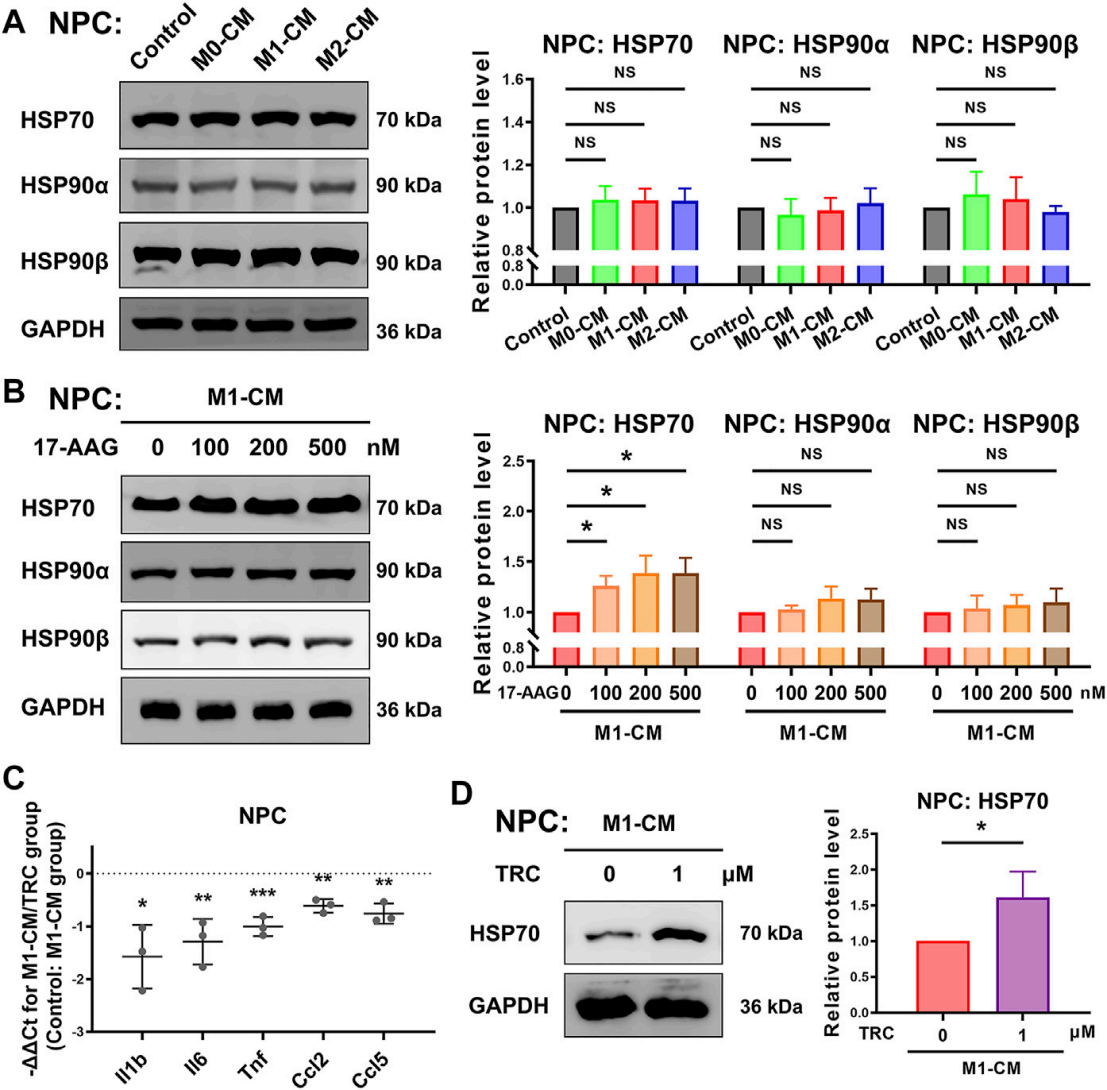
17-AAG induces the expression of HSP70 in NPCs. **(A,B)** Representative WB graphs and statistical analysis of HSP70, HSP90α, HSP90β and GAPDH in NPCs. **(C)** The relative mRNA levels of *Il1b*, *Il6*, *Tnf*, *Ccl2* and *Ccl5* in NPCs from TRC-treated M1-CM group vs. M1-CM group. **(D)** Representative WB graphs and statistical analysis of HSP70 and GAPDH in NPCs. A-D, data were presented as Mean ± SD of three replicates. (*p < 0.05, **p < 0.01, ***p < 0.001. NS, not significant).

WB analysis was conducted to explore the expression of HSP70 and HSP90 in NPCs. As shown in Figure 5A, the expression levels of HSP70, HSP90α and HSP90β in NPCs were not obviously altered by macrophage CM treatment. In M1-CM-stimulated NPCs, administration of 17-AAG dose-dependently increased the expression of HSP70. However, the levels of HSP90α and HSP90β were not dramatically modified by 17-AAG treatment at the doses tested (Figure 5B).

In order to further explore the role of HSP70 in NP inflammation induced by M1-CM, HSP70 activator TRC051384 was used. The results showed that treatment with 1 μM TRC051384 promoted the expression of HSP70, and decreased the expression of *Il1b*, *Il6*, *Tnf*, *Ccl2* and *Ccl5* in NPCs (Figures 5C,D).

### 17-AAG Downregulates the JAK2-STAT3 Pathway Activated by M1-CM

**FIGURE 6:**
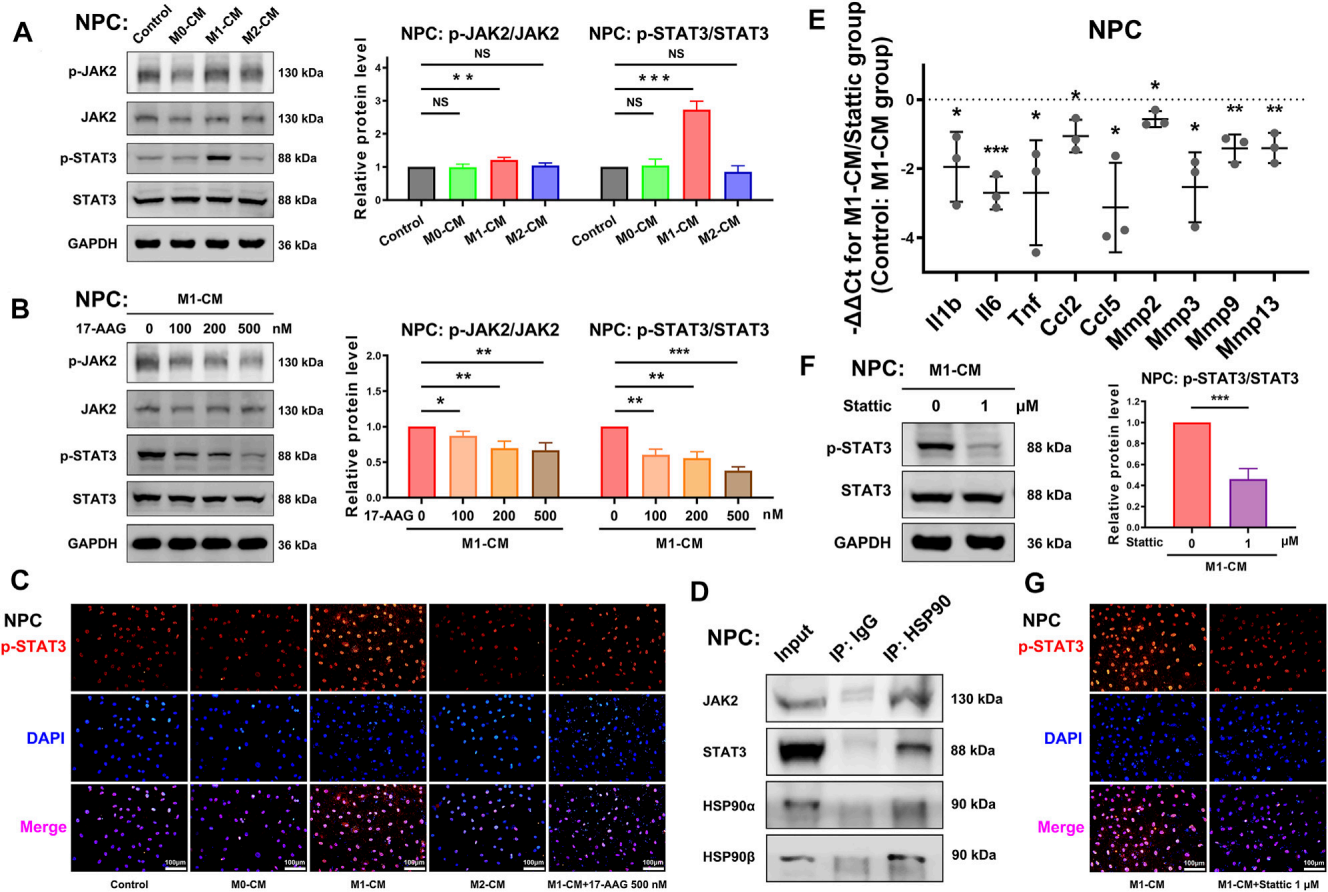
The JAK2-STAT3 pathway is involved in the anti-inflammatory and anti-catabolic effects of 17-AAG. **(A,B)** Representative WB graphs and statistical analysis of p-JAK2, JAK2, p-STAT3, STAT3 and GAPDH in NPCs. **(C)** Typical fluorescence photomicrographs of IF staining for p-STAT3 in NPCs. **(D)** WB graphs of immunoprecipitation using control IgG or anti-HSP90 antibody. **(E)** The relative mRNA levels of *Il1b*, *Il6*, *Tnf*, *Ccl2*, *Ccl5*, *Mmp2*, *Mmp3*, *Mmp9*, and *Mmp13* in NPCs from Stattic-treated M1-CM group vs. M1-CM group. **(F)** Representative WB graphs and statistical analysis of p-STAT3, STAT3 and GAPDH in NPCs. **(G)** Typical fluorescence photomicrographs of IF staining for p-STAT3 in NPCs. A-B, E-F, data were presented as Mean ± SD of three replicates. (*p < 0.05, **p < 0.01, ***p < 0.001. NS, not significant).

The JAK2-STAT3 pathway plays a crucial role in the signalling cascades of multiple cytokines and growth factors. WB analysis showed that p-JAK2/JAK2 ratio and p-STAT3/STAT3 ratio were increased by M1-CM stimulation in NPCs. (Figure 6A) Furthermore, 17-AAG impeded the activation of the JAK2-STAT3 pathway. (Figure 6B) IF staining also showed similar level changes of p-STAT3, which was mainly localized in the nuclei of NPCs. (Figure 6C) Co-immunoprecipitation assay confirmed that JAK2 and STAT3 specifically interacted with molecular chaperone HSP90 (Figure 6D).

A selective STAT3 inhibitor Stattic was used to further investigate the role of STAT3 in the degenerative phenotype of NPCs. PCR showed that 1 μM Stattic suppressed the gene expression of *Il1b*, *Il6*, *Tnf*, *Ccl2*, *Ccl5*, *Mmp2*, *Mmp3*, *Mmp9* and *Mmp13* in NPCs. (Figure 6E) The results of WB analysis (Figure 6F) and IF staining (Figure 6G) supported the inhibiting effects of Stattic on the phosphorylation of STAT3. Taken together, these data implied that the activation of the JAK2-STAT3 pathway was involved in the pro-inflammatory and pro-catabolic effects of M1-CM.

### 17-AAG Attenuates M1-CM-Induced IVD Inflammation and Catabolism in a Murine IVD *in vitro* Culture Model

Experimental organ culture models of IVD could mimic the pro-inflammatory and catabolic microenvironment *in vitro*, providing valuable insights on the mechanisms of IVDD and the explorations of novel therapeutic approaches (Pfannkuche et al., 2020). Our results showed that the expression of inflammatory factors IL-1β and TNF-α, and catabolic protease MMP13 in murine lumbar IVD tissues were upregulated in M1-CM group but not in M2-CM group compared with control group. In presence of 500 nM 17-AAG, all the above protein levels were reduced (Supplementary Figure S4).

### 17-AAG Alleviates IVD Inflammation and Catabolism in a Murine IVDD Model

**FIGURE 7:**
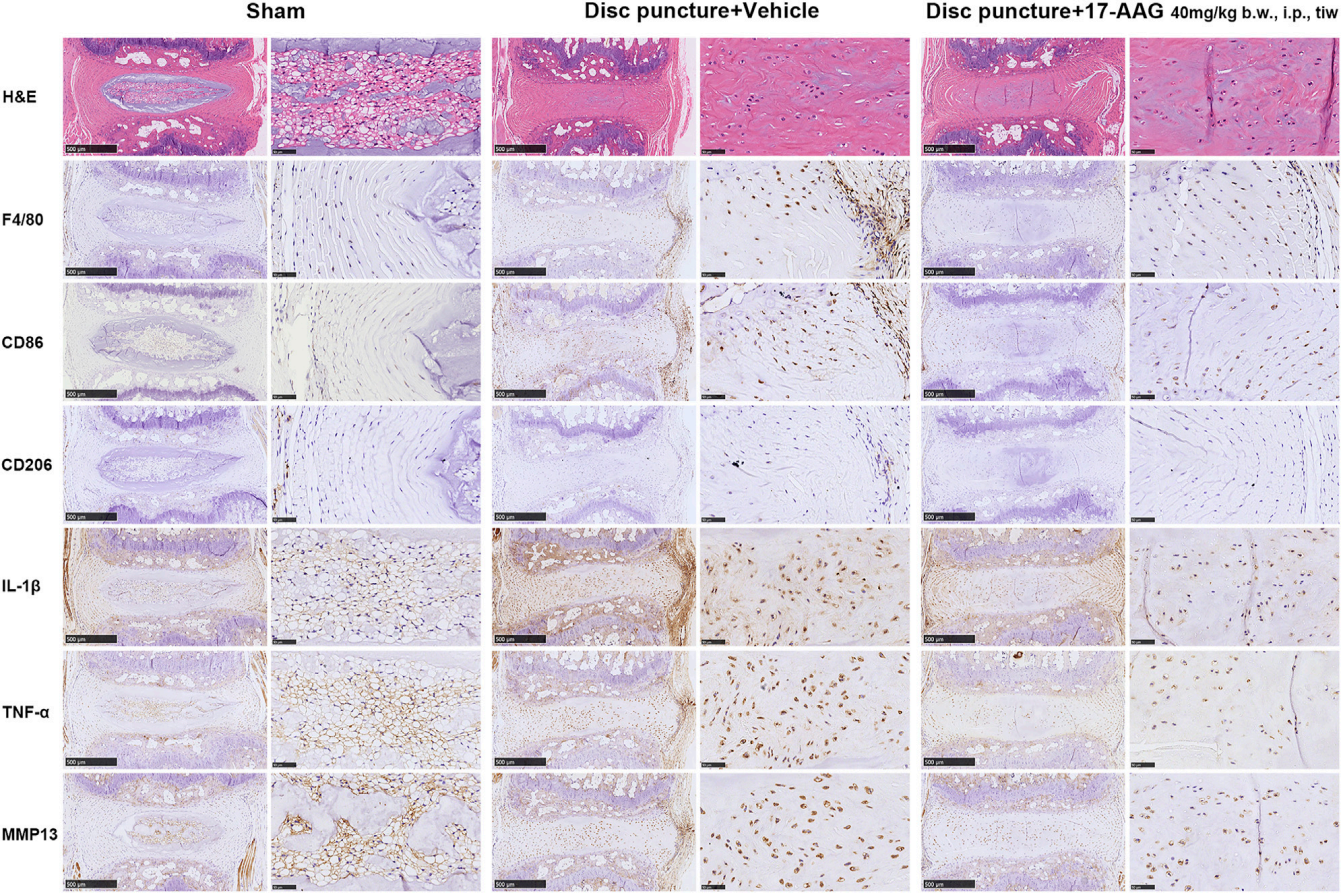
Staining evaluations of murine IVDD models. H&E staining and IHC staining for F4/80, CD86, CD206, IL-1β, TNF-α and MMP13 in coccygeal IVD tissues of murine IVDD models.

To investigate the effects of 17-AAG on the IVDD pathology *in vivo*, we administrated 17-AAG for mice after coccygeal disc puncture. Hematoxylin and eosin (H&E) staining showed loss of IVD height and disruption of IVD structure in disc puncture group. IHC staining showed that macrophages stained by F4/80 infiltrated through annulus fibrosus (AF) lamellae surrounding the puncture tract, while 17-AAG treatment prevented the macrophage infiltration into degenerated IVDs. Of the infiltrated macrophage population, CD86-positive M1 macrophages were more abundant than CD206-positive M2 macrophages. Moreover, the levels of IL-1β, TNF-α and MMP13 were elevated in both NP and AF regions in disc puncture/vehicle groups. 17-AAG treatment downregulated the levels of IL-1β, TNF-α and MMP13, signifying the anti-inflammatory and anti-catabolic effects of 17-AAG *in vivo* (Figure 7).

## Discussion

**FIGURE 8:**
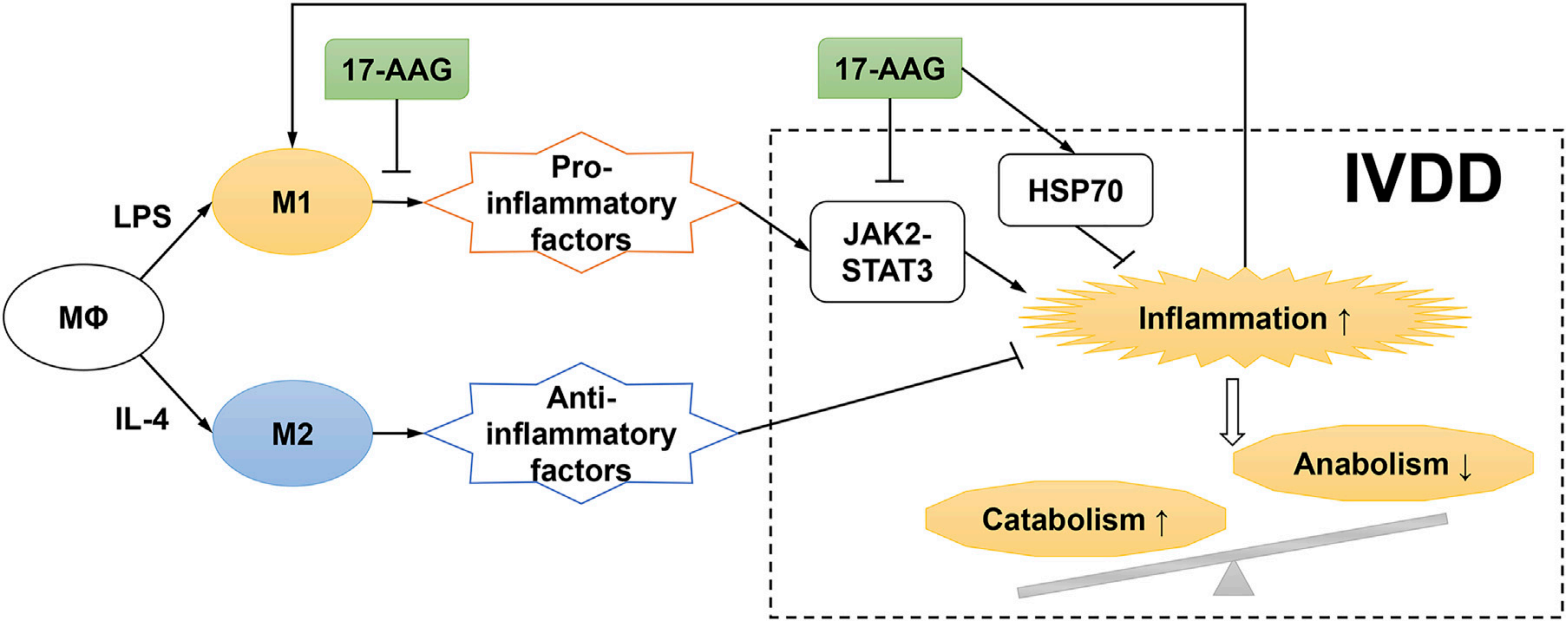
Schematic diagram for proposed mechanisms of the effects of 17-AAG on NP inflammation and catabolism induced by M1-polarized macrophages.

IVDD, a common but complex process, is frequently associated with LBP. Evidence showed that the pro-inflammatory microenvironment played a critical role in IVDD progression and LBP development (Zhang et al., 2021a; Lyu et al., 2021). Multiple anti-cytokine agents and signaling pathway inhibitors were investigated to ameliorate IVD inflammation and delay IVDD in preclinical and clinical studies (Risbud et al., 2014). The current study demonstrated that IVD-infiltrated inflammatory macrophages promoted the inflammation and catabolism in degenerated IVDs. Moreover, the anti-inflammatory and anti-catabolic effects of 17-AAG were also addressed (Figure 8).

Under microenvironmental stimuli, circulating monocytes were recruited into multiple tissues and differentiated into macrophages. IHC staining of macrophage markers in human disc tissues indicated that the frequency of macrophages markedly increased with degenerative grade (Shamji et al., 2010; Nakazawa et al., 2018). The following two pathological processes of IVDD might be involved in the infiltration of macrophages. On the one hand, ECM degradation during IVDD resulted in structural defects and blood vessels ingrowth, which facilitate the macrophage infiltration into degenerated IVD tissues (Kobayashi et al., 2009; Dongfeng et al., 2011). On the other hand, degenerated IVD is characterized by inflammatory microenvironment, involving the accumulation of senescent cells and damage-associated molecular patterns (Wang et al., 2016; Bisson et al., 2021). Inflammatory microenvironment may also induce the polarization of infiltrated macrophages towards inflammatory phenotype. Additionally, elevated levels of inflammatory and chemotactic mediators produced by IVD cells and infiltrated macrophages further promoted the recruitment of macrophages, which might aggravate the positive feedback loop of inflammation activation (Yoshida et al., 2005).

Pro-inflammatory macrophages are highly involved in the development of musculoskeletal diseases, such as osteoporosis and arthritis. Inflammatory factors derived from activated macrophages exert pro-inflammatory and destructive effects on bone and cartilage tissues (Rachner et al., 2011; Wu et al., 2020). The current study indicated that M1 macrophages and related inflammatory cascades also played an essential role in the pathological process of IVDD. Macrophages in or adjacent to IVD tissues may secrete various pro-inflammatory cytokines such as IL-1β, TNF-α, IL-6 and IL-8, thus exacerbating the inflammatory phenotype of NPCs. Additionally, M1 macrophages activate NPCs to release destructive enzymes, which might break down disc ECM components such as proteoglycans and collagens. Our findings are consistent with previous study which reported that pro-inflammatory M1 macrophages promoted the degenerative phenotypes of IVD cells (Ni et al., 2019; Yang et al., 2019).

Macrophages retain remarkable plasticity, and phenotype switch may occur over time in response to environmental signals. Deleting whole macrophages without discriminating the subtypes seems to be an arbitrary approach to alleviate tissue inflammation (Wu et al., 2017). Emerging evidence supported that the orchestration of M1/M2 macrophage populations played a critical role in tissue homeostasis. IL-4-treated macrophages could assume a wound-healing phenotype, which facilitate matrix reorganization and tissue repair, whereas, possess poorer pro-inflammation and anti-infection abilities than M1 macrophages (Mosser and Edwards, 2008). Thus, a hypothesis emerged that moderately activated wound-healing macrophages may be conducive to the tissue repair of degenerated IVD. Our results indicated that M2-CM did not promote NP inflammation and catabolism, while the anti-inflammatory and matrix-enhancing activities of M2 macrophages were not systematically investigated.

HSP90 is a highly conserved and widely expressed chaperone family, which modulates the maturation and stabilization of intracellular proteins involved in signal transduction and transcriptional regulation. Inhibiting HSP90 is recognized as a promising therapeutic strategy for multiple inflammatory diseases by upregulating HSP70 expression, mediating HSP90 client proteins degradation and dampening inflammatory signaling cascades. In inflammatory macrophages, HSP90 inhibitors EC144 and 17-DMAG could diminish the sensitization of macrophages to LPS, and decrease the release of inflammatory factors *via* inhibiting the MAPK, NF-κB and NOD-like receptor protein 3 (NLRP3) pathways (Yun et al., 2011; Ambade et al., 2014; Nizami et al., 2021). Our results revealed the similar effects of 17-AAG on the M1 polarization of macrophages. Moreover, HSP90 inhibitor 17-AAG could exert anti-inflammatory effects, and control the progression of diverse inflammatory diseases, such as autoimmune dermatitis (Tukaj et al., 2017), uveitis (Poulaki et al., 2007), etc. The current study demonstrated that 17-AAG dose-dependently restored NP inflammation and catabolism by simultaneously ameliorating the inflammatory phenotype of macrophages and NPCs. Our previous researches reported that HSP90 inhibitor BIIB021 and HSP70 activator TRC051384 could protect NPSCs viability and function via preventing mitochondrial dysfunction (Hu et al., 2020; Zhang et al., 2021b). Similarly, the protective effects of 17-AAG on NPCs were detected in our ongoing experiments, which further brightened the application prospects of 17-AAG in IVDD treatment.

The JAK2-STAT3 pathway is discovered as a critical part of cytokine signalling cascades, activated by a multitude of inflammatory cytokines, growth factors and peptide hormones, beyond IL-6 family members (Bharadwaj et al., 2020). Phosphorylated by JAK2, dimerized p-STAT3 enters the nucleus, and works as a transcription factor to initiate the expression of multiple genes and regulate downstream signaling pathways (Huynh et al., 2019). Inhibiting STAT3 is an effective strategy to treat chronic inflammation diseases including osteoarthritis (Latourte et al., 2017), muscle wasting (Tierney et al., 2014), etc*.* The STAT3 pathway was also involved in IL-6-stimulated inflammatory and catabolic phenotype of AF cells (Suzuki et al., 2017). JAK2 and STAT3 are clients of the molecular chaperone HSP90, and HSP90 tightly regulates the activation of JAK2-STAT3 signalling (Marubayashi et al., 2010; Serrano-Marco et al., 2011). Combination with HSP90 inhibitors is evaluated as an effective strategy to overcome the resistance of JAK2 inhibitors in fibrotic diseases and myeloproliferative neoplasms (Zhang et al., 2017). Correspondingly, our results showed that 17-AAG prevented M1-CM-induced IVD inflammation via inhibiting the aberrant activation of the JAK2-STAT3 pathway.

Several crucial but unsolved problems need to be further elucidated in the subsequent researches. Firstly, the classical M1/M2 subdivision hindered the understanding of macrophage plasticity, and several subtypes of M2 macrophages (M2a, M2b, M2c, and M2d) were recently categorized based on activating approaches and secreted cytokine profiles (Xue et al., 2014). Moreover, the temporal changes of macrophage phenotypes during IVD injury and repair required to be further investigated. Secondly, tissue-resident macrophages, a self-renewable cell population independently of circulating monocytes, developed from embryonic hematopoiesis and colonized most organs during embryogenesis (Ginhoux et al., 2016)*.* Tissue-resident macrophages play a pivotal role in tissue homeostasis, inflammation and defense (Davies et al., 2013). However, the existence and nature of tissue-resident macrophages or macrophage-like cells in IVD were not well explored (Kawakubo et al., 2020). Thirdly, resident IVD cells might also express macrophage cell markers, such as CD68 (Jones et al., 2008). Hence, further efforts are needed to design more specific methodologies to detect macrophage populations in IVD.

Several limitations of the current research should be underlined. Firstly, our study addressed the detrimental role of M1 macrophages in severely degenerated IVD tissues. However, the mechanisms of the initiation of IVDD were not elucidated. Secondly, the number of mildly degenerated human IVD samples was relatively small due to the difficulty of obtaining Grade I-II discs.

## Conclusion

M1 macrophages could secrete multiple destructive factors and promote degenerative changes of IVD. Moreover, HSP90 inhibitor 17-AAG represents therapeutic effects by not only attenuating the pro-inflammatory phenotype of M1 macrophages but also dampening NP inflammation and catabolism.

## Data Availability

The original contributions presented in the study are included in the article or Supplementary Material. Further inquiries can be directed to the corresponding author.

## References

[B1] Aliverti E. Tilson J. L. Filer D. L. Babcock B. Colaneri A. Ocasio J. (2020). Projected t-SNE for Batch Correction. Bioinformatics (Oxford, England) 36, 3522–3527. 10.1093/bioinformatics/btaa189 PMC7267829 32176244

[B2] Ambade A. Catalano D. Lim A. Kopoyan A. Shaffer S. A. Mandrekar P. (2014). Inhibition of Heat Shock Protein 90 Alleviates Steatosis and Macrophage Activation in Murine Alcoholic Liver Injury. J. Hepatol. 61, 903–911. 10.1016/j.jhep.2014.05.024 24859453 PMC4169725

[B3] Bharadwaj U. Kasembeli M. M. Robinson P. Tweardy D. J. (2020). Targeting Janus Kinases and Signal Transducer and Activator of Transcription 3 to Treat Inflammation, Fibrosis, and Cancer: Rationale, Progress, and Caution. Pharmacol. Rev. 72, 486–526. 10.1124/pr.119.018440 32198236 PMC7300325

[B4] Bisson D. Mannarino M. Mannarino M. Racine R. Haglund L. (2021). For Whom the Disc Tolls: Intervertebral Disc Degeneration, Back Pain and Toll-like Receptors. eCM 41, 355–369. 10.22203/eCM.v041a23 33738788

[B5] Butler A. Hoffman P. Smibert P. Papalexi E. Satija R. (2018). Integrating Single-Cell Transcriptomic Data across Different Conditions, Technologies, and Species. Nat. Biotechnol. 36, 411–420. 10.1038/nbt.4096 29608179 PMC6700744

[B6] Davies L. C. Jenkins S. J. Allen J. E. Taylor P. R. (2013). Tissue-resident Macrophages. Nat. Immunol. 14, 986–995. 10.1038/ni.2705 24048120 PMC4045180

[B7] Dongfeng R. Hou S. Wu W. Wang H. Shang W. Tang J. (2011). The Expression of Tumor Necrosis Factor-α and CD68 in High-Intensity Zone of Lumbar Intervertebral Disc on Magnetic Resonance Image in the Patients with Low Back Pain. Spine 36, E429–E433. 10.1097/BRS.0b013e3181dfce9e 21192298

[B8] Ginhoux F. Guilliams M. (2016). Tissue-Resident Macrophage Ontogeny and Homeostasis. Immunity 44, 439–449. 10.1016/j.immuni.2016.02.024 26982352

[B9] Hu B. Zhang S. Liu W. Wang P. Chen S. Lv X. (2020). Inhibiting Heat Shock Protein 90 Protects Nucleus Pulposus-Derived Stem/Progenitor Cells from Compression-Induced Necroptosis and Apoptosis. Front. Cel Dev. Biol. 8, 685. 10.3389/fcell.2020.00685 PMC7427414 32850811

[B10] Huynh J. Chand A. Gough D. Ernst M. (2019). Therapeutically Exploiting STAT3 Activity in Cancer - Using Tissue Repair as a Road Map. Nat. Rev. Cancer 19, 82–96. 10.1038/s41568-018-0090-8 30578415

[B11] Jones P. Gardner L. Menage J. Williams G. T. Roberts S. (2008). Intervertebral Disc Cells as Competent Phagocytes *In Vitro*: Implications for Cell Death in Disc Degeneration. Arthritis Res. Ther. 10, R86. 10.1186/ar2466 18673547 PMC2575634

[B12] Kawakubo A. Uchida K. Miyagi M. Nakawaki M. Satoh M. Sekiguchi H. (2020). Investigation of Resident and Recruited Macrophages Following Disc Injury in Mice. J. Orthop. Res. 38, 1703–1709. 10.1002/jor.24590 31965590

[B13] Khan A. N. Jacobsen H. E. Khan J. Filippi C. G. Levine M. Lehman R. A. Jr. (2017). Inflammatory Biomarkers of Low Back Pain and Disc Degeneration: a Review. Ann. N.Y. Acad. Sci. 1410, 68–84. 10.1111/nyas.13551 29265416 PMC5744892

[B14] Kobayashi S. Meir A. Kokubo Y. Uchida K. Takeno K. Miyazaki T. (2009). Ultrastructural Analysis on Lumbar Disc Herniation Using Surgical Specimens. Spine 34, 655–662. 10.1097/BRS.0b013e31819c9d5b 19333096

[B15] Latourte A. Cherifi C. Maillet J. Ea H.-K. Bouaziz W. Funck-Brentano T. (2017). Systemic Inhibition of IL-6/Stat3 Signalling Protects against Experimental Osteoarthritis. Ann. Rheum. Dis. 76, 748–755. 10.1136/annrheumdis-2016-209757 27789465

[B16] Liu J. W. Lin K. H. Weber C. Bhalla S. Kelso S. Wang K. (2017). An *In Vitro* Organ Culture Model of the Murine Intervertebral Disc. JoVE 11, 55437. 10.3791/55437 PMC5495460 28448052

[B17] Lyu F.-J. Cui H. Pan H. Mc Cheung K. Cao X. Iatridis J. C. (2021). Painful Intervertebral Disc Degeneration and Inflammation: from Laboratory Evidence to Clinical Interventions. Bone Res. 9, 7. 10.1038/s41413-020-00125-x 33514693 PMC7846842

[B18] Marubayashi S. Koppikar P. Taldone T. Abdel-Wahab O. West N. Bhagwat N. (2010). HSP90 Is a Therapeutic Target in JAK2-dependent Myeloproliferative Neoplasms in Mice and Humans. J. Clin. Invest. 120, 3578–3593. 10.1172/jci42442 20852385 PMC2947224

[B19] Mosser D. M. Edwards J. P. (2008). Exploring the Full Spectrum of Macrophage Activation. Nat. Rev. Immunol. 8, 958–969. 10.1038/nri2448 19029990 PMC2724991

[B20] Murray P. J. Wynn T. A. (2011). Protective and Pathogenic Functions of Macrophage Subsets. Nat. Rev. Immunol. 11, 723–737. 10.1038/nri3073 21997792 PMC3422549

[B21] Nakazawa K. R. Walter B. A. Laudier D. M. Krishnamoorthy D. Mosley G. E. Spiller K. L. (2018). Accumulation and Localization of Macrophage Phenotypes with Human Intervertebral Disc Degeneration. Spine J. 18, 343–356. 10.1016/j.spinee.2017.09.018 29031872 PMC5815908

[B22] Ni L. Zheng Y. Gong T. Xiu C. Li K. Saijilafu (2019). Proinflammatory Macrophages Promote Degenerative Phenotypes in Rat Nucleus Pulpous Cells Partly through ERK and JNK Signaling. J. Cel Physiol 234, 5362–5371. 10.1002/jcp.27507 30367477

[B23] Nizami S. Arunasalam K. Green J. Cook J. Lawrence C. B. Zarganes‐Tzitzikas T. (2021). Inhibition of the NLRP3 Inflammasome by HSP90 Inhibitors. Immunology 162, 84–91. 10.1111/imm.13267 32954500 PMC7730016

[B24] Pfannkuche J.-J. Guo W. Cui S. Ma J. Lang G. Peroglio M. (2020). Intervertebral Disc Organ Culture for the Investigation of Disc Pathology and Regeneration - Benefits, Limitations, and Future Directions of Bioreactors. Connect. Tissue Res. 61, 304–321. 10.1080/03008207.2019.1665652 31556329

[B25] Pfirrmann C. W. A. Metzdorf A. Zanetti M. Hodler J. Boos N. (2001). Magnetic Resonance Classification of Lumbar Intervertebral Disc Degeneration. Spine 26, 1873–1878. 10.1097/00007632-200109010-00011 11568697

[B26] Poulaki V. Iliaki E. Mitsiades N. Mitsiades C. S. Paulus Y. N. Bula D. V. (2007). Inhibition of Hsp90 Attenuates Inflammation in Endotoxin‐induced Uveitis. FASEB j. 21, 2113–2123. 10.1096/fj.06-7637com 17400913

[B27] Rachner T. D. Khosla S. Hofbauer L. C. (2011). Osteoporosis: Now and the Future. The Lancet 377, 1276–1287. 10.1016/s0140-6736(10)62349-5 PMC3555696 21450337

[B28] Rice J. W. Veal J. M. Fadden R. P. Barabasz A. F. Partridge J. M. Barta T. E. (2008). Small Molecule Inhibitors of Hsp90 Potently Affect Inflammatory Disease Pathways and Exhibit Activity in Models of Rheumatoid Arthritis. Arthritis Rheum. 58, 3765–3775. 10.1002/art.24047 19035474

[B29] Risbud M. V. Shapiro I. M. (2014). Role of Cytokines in Intervertebral Disc Degeneration: Pain and Disc Content. Nat. Rev. Rheumatol. 10, 44–56. 10.1038/nrrheum.2013.160 24166242 PMC4151534

[B30] Schmittgen T. D. Livak K. J. (2008). Analyzing Real-Time PCR Data by the Comparative CT Method. Nat. Protoc. 3, 1101–1108. 10.1038/nprot.2008.73 18546601

[B31] Schopf F. H. Biebl M. M. Buchner J. (2017). The HSP90 Chaperone Machinery. Nat. Rev. Mol. Cel Biol 18, 345–360. 10.1038/nrm.2017.20 28429788

[B32] Serrano-Marco L. Rodríguez-Calvo R. El Kochairi I. Palomer X. Michalik L. Wahli W. (2011). Activation of Peroxisome Proliferator-Activated Receptor-/- (PPAR-/- ) Ameliorates Insulin Signaling and Reduces SOCS3 Levels by Inhibiting STAT3 in Interleukin-6-Stimulated Adipocytes. Diabetes 60, 1990–1999. 10.2337/db10-0704 21617181 PMC3121427

[B33] Shamji M. F. Setton L. A. Jarvis W. So S. Chen J. Jing L. (2010). Pro-inflammatory Cytokine Expression Profile in Degenerative and Herniated Human Intervertebral Disc Tissues. Arthritis Rheum. 62, 27444. 10.1002/art.27444 PMC2917579 20222111

[B34] Siebelt M. Jahr H. Groen H. C. Sandker M. Waarsing J. H. Kops N. (2013). Hsp90 Inhibition Protects against Biomechanically Induced Osteoarthritis in Rats. Arthritis Rheum. 65, 2102–2112. 10.1002/art.38000 23666904

[B35] Suzuki S. Fujita N. Fujii T. Watanabe K. Yagi M. Tsuji T. (2017). Potential Involvement of the IL-6/JAK/STAT3 Pathway in the Pathogenesis of Intervertebral Disc Degeneration. Spine 42, E817–E824. 10.1097/brs.0000000000001982 27879577

[B36] Tierney M. T. Aydogdu T. Sala D. Malecova B. Gatto S. Puri P. L. (2014). STAT3 Signaling Controls Satellite Cell Expansion and Skeletal Muscle Repair. Nat. Med. 20, 1182–1186. 10.1038/nm.3656 25194572 PMC4332844

[B37] Tukaj S. Bieber K. Kleszczyński K. Witte M. Cames R. Kalies K. (2017). Topically Applied Hsp90 Blocker 17AAG Inhibits Autoantibody-Mediated Blister-Inducing Cutaneous Inflammation. J. Invest. Dermatol. 137, 341–349. 10.1016/j.jid.2016.08.032 27659253

[B38] Tukaj S. Węgrzyn G. (2016). Anti-Hsp90 Therapy in Autoimmune and Inflammatory Diseases: a Review of Preclinical Studies. Cell Stress and Chaperones 21, 213–218. 10.1007/s12192-016-0670-z 26786410 PMC4786535

[B39] Wang F. Cai F. Shi R. Wang X.-H. Wu X.-T. (2016). Aging and Age Related Stresses: a Senescence Mechanism of Intervertebral Disc Degeneration. Osteoarthritis and cartilage 24, 398–408. 10.1016/j.joca.2015.09.019 26455958

[B40] Wang J. Huang Y. Huang L. Shi K. Wang J. Zhu C. (2021). Novel Biomarkers of Intervertebral Disc Cells and Evidence of Stem Cells in the Intervertebral Disc. Osteoarthritis and Cartilage 29, 389–401. 10.1016/j.joca.2020.12.005 33338640

[B41] Wu C.-L. Harasymowicz N. S. Klimak M. A. Collins K. H. Guilak F. (2020). The Role of Macrophages in Osteoarthritis and Cartilage Repair. Osteoarthritis and cartilage 28, 544–554. 10.1016/j.joca.2019.12.007 31926267 PMC7214213

[B42] Wu C.-L. McNeill J. Goon K. Little D. Kimmerling K. Huebner J. (2017). Conditional Macrophage Depletion Increases Inflammation and Does Not Inhibit the Development of Osteoarthritis in Obese Macrophage Fas-Induced Apoptosis-Transgenic Mice. Arthritis Rheumatol. 69, 1772–1783. 10.1002/art.40161 28544542 PMC5611814

[B43] Xue J. Schmidt S. V. Sander J. Draffehn A. Krebs W. Quester I. (2014). Transcriptome-based Network Analysis Reveals a Spectrum Model of Human Macrophage Activation. Immunity 40, 274–288. 10.1016/j.immuni.2014.01.006 24530056 PMC3991396

[B44] Yang H. Liu B. Liu Y. He D. Xing Y. An Y. (2019). Secreted Factors from Intervertebral Disc Cells and Infiltrating Macrophages Promote Degenerated Intervertebral Disc Catabolism. Spine 44, E520–E529. 10.1097/brs.0000000000002953 30540714

[B45] Yoshida M. Nakamura T. Sei A. Kikuchi T. Takagi K. Matsukawa A. (2005). Intervertebral Disc Cells Produce Tumor Necrosis Factor α, Interleukin-1β, and Monocyte Chemoattractant Protein-1 Immediately after Herniation: An Experimental Study Using a New Hernia Model. Spine 30, 55–61. 10.1097/01.brs.0000149194.17891.bf 15626982

[B46] Yun T. J. Harning E. K. Giza K. Rabah D. Li P. Arndt J. W. (2011). EC144, a Synthetic Inhibitor of Heat Shock Protein 90, Blocks Innate and Adaptive Immune Responses in Models of Inflammation and Autoimmunity. J.Immunol. 186, 563–575. 10.4049/jimmunol.1000222 21131419

[B47] Zhang S. Hu B. Liu W. Wang P. Lv X. Chen S. (2021a). The Role of Structure and Function Changes of Sensory Nervous System in Intervertebral Disc-Related Low Back Pain. Osteoarthritis and cartilage 29, 17–27. 10.1016/j.joca.2020.09.002 33007412

[B48] Zhang S. Liu W. Wang P. Hu B. Lv X. Chen S. (2021b). Activation of HSP70 Impedes Tert-Butyl Hydroperoxide (t-Bhp)-Induced Apoptosis and Senescence of Human Nucleus Pulposus Stem Cells via Inhibiting the JNK/c-Jun Pathway. Mol. Cel Biochem 476, 1979–1994. 10.1007/s11010-021-04052-1 33511552

[B49] Zhang X. Lan Y. Xu J. Quan F. Zhao E. Deng C. (2019). CellMarker: a Manually Curated Resource of Cell Markers in Human and Mouse. Nucleic Acids Res. 47, D721–D728. 10.1093/nar/gky900 30289549 PMC6323899

[B50] Zhang Y. Liang R. Chen C.-W. Mallano T. Dees C. Distler A. (2017). JAK1-dependent Transphosphorylation of JAK2 Limits the Antifibrotic Effects of Selective JAK2 Inhibitors on Long-Term Treatment. Ann. Rheum. Dis. 76, 1467–1475. 10.1136/annrheumdis-2016-210911 28478401

[B51] Zhou F. Mei J. Han X. Li H. Yang S. Wang M. (2019). Kinsenoside Attenuates Osteoarthritis by Repolarizing Macrophages through Inactivating NF-ΚB/MAPK Signaling and Protecting Chondrocytes. Acta pharmaceutica Sinica B 9, 973–985. 10.1016/j.apsb.2019.01.015 31649847 PMC6804452

