# Supplementary material for: HSP90 Inhibitor 17-AAG Attenuates Nucleus Pulposus Inflammation and Catabolism Induced by M1-Polarized Macrophages
Source: Front Cell Dev Biol. 2022 Jan 4;9:796974. doi: 10.3389/fcell.2021.796974 (PMC8763810; doi:10.3389/fcell.2021.796974)
Supplement: Supplementary file 1 [file Table1.DOCX]

Supplementary Material

# Supplementary Tables

**Supplementary Table S1** Characteristics of the patients enrolled in the study

| **Case Number** | **Age (Years)** | **Gender** | **Diagnosis** | **Disc segment** | **Pfirrmann Grade** |
| --- | --- | --- | --- | --- | --- |
| 1 | 58 | M | LDH | L4/5 | Ⅳ |
| 2 | 54 | F | LDH | L4/5 | Ⅳ |
| 3 | 46 | F | LDH | L3/4 | Ⅱ |
| 4 | 57 | F | LDH | L4/5 | Ⅱ |
| 5 | 35 | F | LDH | L5/S1 | Ⅴ |
| 6 | 56 | M | LDH | L5/S1 | Ⅲ |
| 7 | 41 | F | LDH | L4/5 | Ⅳ |
| 8 | 63 | F | LDH | L4/5 | Ⅲ |
| 9 | 55 | F | LDH | L4/5 | Ⅱ |
| 10 | 53 | F | LDH | L3/4 | Ⅲ |
| 11 | 47 | F | LDH | L3/4 | Ⅳ |
| 12 | 43 | M | LDH | L5/S1 | Ⅳ |

LDH: lumbar disc herniation

**Supplementary Table S2** The primer sequences for qRT-PCR used in the study

| **Gene** | **NCBI Gene ID** | **Forward primer (5' to 3')** | **Reverse primer (5' to 3')** |
| --- | --- | --- | --- |
| M-*Gapdh* | 14433 | GACAAAATGGTGAAGGTCGGT | GAGGTCAATGAAGGGGTCG |
| M-*Il1b* | 16176 | ATGGGCTGGACTGTTTCTAATG | CTTGTGACCCTGAGCGACC |
| M-*Il4* | 16189 | TCAACCCCCAGCTAGTTGTC | TGTTCTTCGTTGCTGTGAGG |
| M-*Il6* | 16193 | AGTTGCCTTCTTGGGACTGA | TCCACGATTTCCCAGAGAAC |
| M-*Il8* | 20309 | CCCGCGTTAGTCTGGTGTAT | AACAGCCCATAGTGGAGTGG |
| M-*Tnf* | 21926 | CGGGCAGGTCTACTTTGGAG | ACCCTGAGCCATAATCCCCT |
| M-*Nos2* | 18126 | CCTGTGTTCCACCAGGAGAT | CCCTGGCTAGTGCTTCAGAC |
| M-*Mrc1* | 17533 | CCAAAGCTGACCAAAGGAAG | GCCCATGAGATCTTTCGTGT |
| R-*Gapdh* | 24383 | ACAGCAACAGGGTGGTGGAC | TTTGAGGGTGCAGCGAACTT |
| R-*Il1b* | 24494 | GGTGGTTCAAGGCATAACA | TGTCGAGATGCTGCTGTGAG |
| R-*Il6* | 24498 | GACCAAGACCATCCAACT | TAGGTTTGCCGAGTAGAC |
| R-*Tnf* | 24835 | CCAATCTGTGTCCTTCTAA | TTCTGAGCATCGTAGTTG |
| R-*Ccl2* | 24770 | ATGCAGTTAATGCCCCACTC | TTCCTTATTGGGGTCAGCAC |
| R-*Ccl5* | 81780 | ATATGGCTCGGACACCACTC | CAAAGACGACTGCAAGGTTGG |
| R-*Mmp2* | 81686 | CGGGCCGTACAATCTTCACT | AAGTAGCACCTGGGAGGGAT |
| R-*Mmp3* | 171045 | TTTGGCCGTCTCTTCCATCC | GCATCGATCTTCTGGACGGT |
| R-*Mmp9* | 81687 | GATCCCCAGAGCGTTACTCG | GTTGTGGAAACTCACACGCC |
| R-*Mmp13* | 171052 | GGACTCACTGTTGGTCCCTG | GGATTCCCGCAAGAGTCACA |
| R-*Adamts4* | 66015 | GGGGCAGGGTTAAAGCACTA | TTGACAGGGTTTCGGATGCT |
| R-*Adamts5* | 304135 | TCCTCTTGGTGGCTGACTCT | ACCACTTTCACTACGGCCAG |
| R-*Col2a1* | 25412 | CTCAAGTCGCTGAACAACCA | GTCTCCGCTCTTCCACTCTG |

M: mouse; R: rat

**Supplementary Table S3** The antibodies used in the study

| **Antibodies** | **Source** | **Catalog No.** |
| --- | --- | --- |
| GAPDH | Affinity Biosciences | AF7021 |
| JNK | Cell Signaling Technology | 9252T |
| p-JNK (T183/Y185) | Cell Signaling Technology | 4668T |
| ERK | Cell Signaling Technology | 4695T |
| p-ERK (T202/Y204) | Cell Signaling Technology | 4370T |
| p38 MAPK | Abclonal Technology | A10832 |
| p-p38 MAPK (T180/Y182) | Abclonal Technology | AP0526 |
| NF-κB p65 | Abclonal Technology | A10609 |
| p-NF-κB p65 (S529) | Abclonal Technology | AP0944 |
| MMP3 | Proteintech | 66338-1-Ig |
| MMP9 | Abclonal Technology | A2095 |
| MMP13 | Proteintech | 18165-1-AP |
| JAK2 | Abclonal Technology | A11497 |
| p-JAK2 (Y1007/1008) | Abclonal Technology | AP0531 |
| STAT3 | Abclonal Technology | A1192 |
| p-STAT3 (Y705) | Abclonal Technology | AP0070 |
| CD68 | Abcam | ab201340 |
| F4/80 (EMR1) | Abclonal Technology | A1256 |
| CD86 | Abclonal Technology | A1199 |
| MRC1 (CD206) | Abclonal Technology | A8301 |
| IL-1β | Abclonal Technology | A16288 |
| TNF-α | Abclonal Technology | A0277 |
| HSP70 | Abclonal Technology | A12948 |
| HSP90α/β | Santa Cruz Biotechnology. | sc-13119 |
| HSP90α | Abclonal Technology | A12448 |
| HSP90β | Abclonal Technology | A1087 |
| Collagen Ⅱ | Affinity Biosciences | AF0135 |

# Supplementary Figures

**
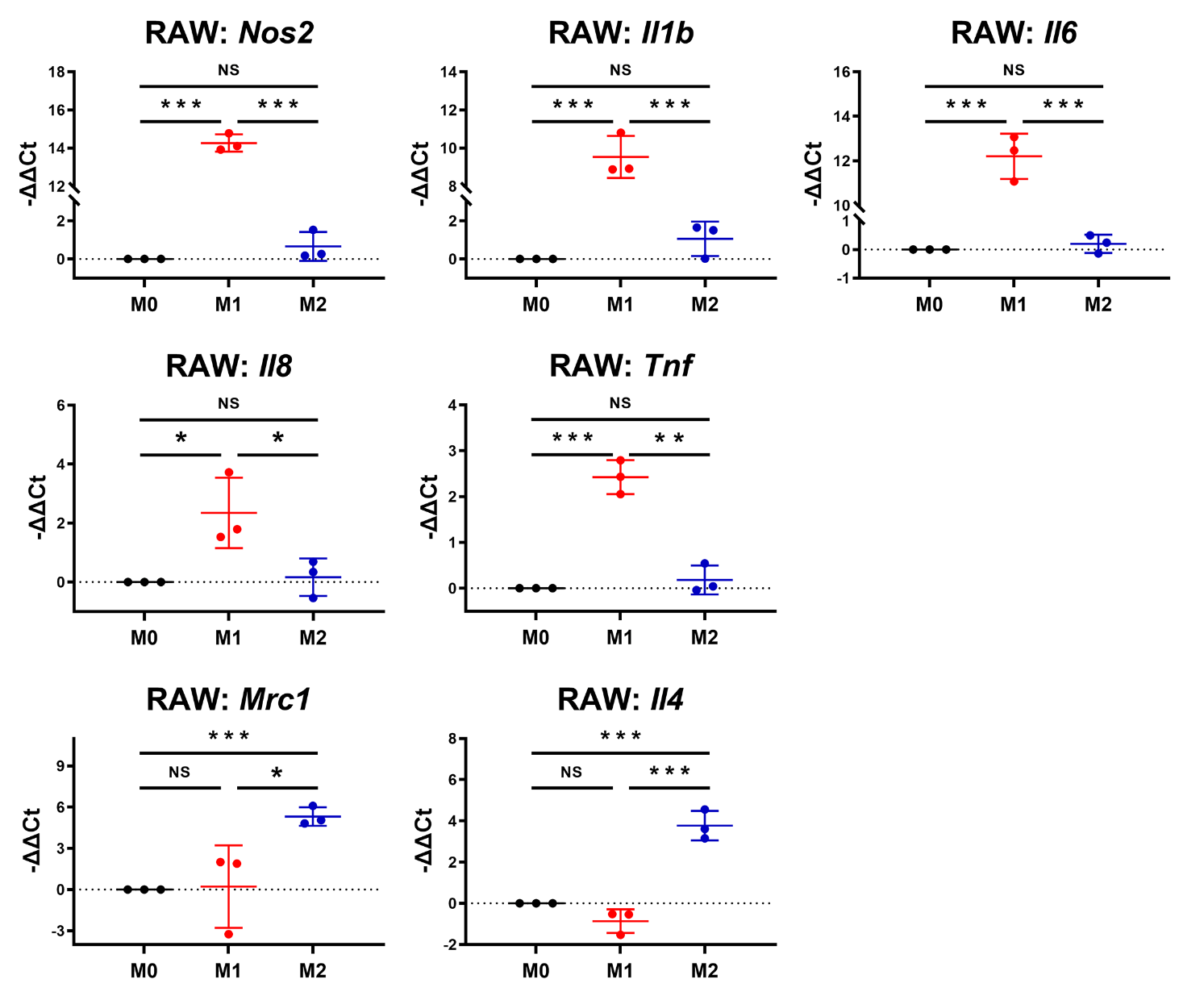
**

**Supplementary Figure S1.** **Gene expression profiles of polarized RAWs**

The mRNA levels of *Nos2*, *Il1b*, *Il6*, *Il8*, *Tnf*, *Mrc1* and *Il4* in RAWs. Data were presented as Mean ± SD of three repeated experiments. (**P*<0.05, ***P*<0.01, ****P*<0.001. NS, not significant)


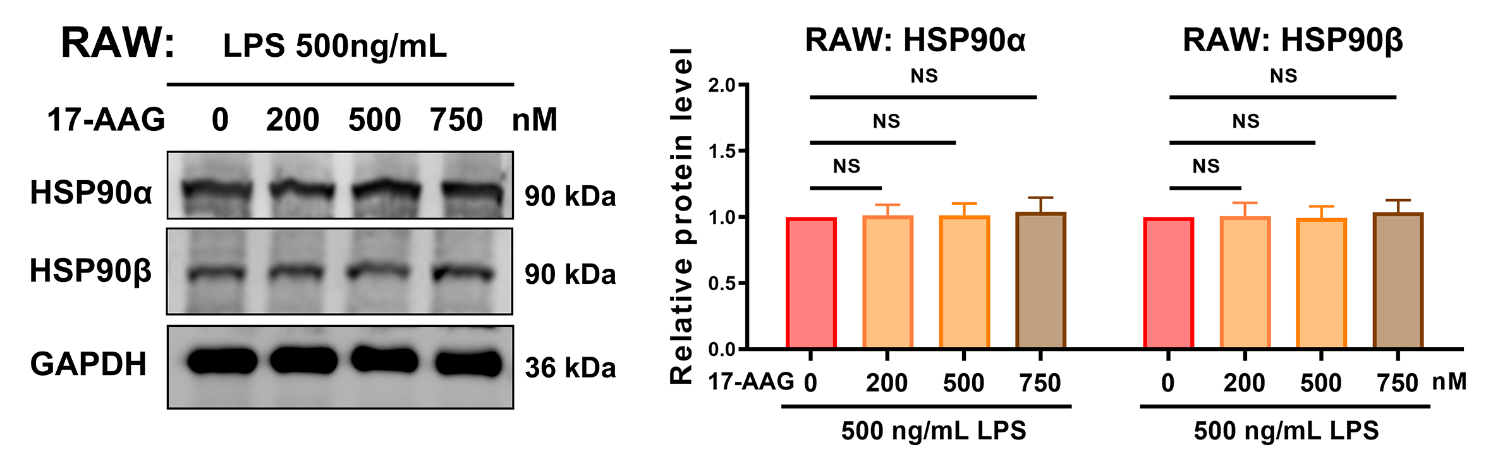


**Supplementary Figure S2. Effects of 17-AAG on HSP90 expression in RAWs**

Representative WB graphs and statistical analysis of HSP90α, HSP90β and GAPDH in RAWs. Data were presented as Mean ± SD of three repeated experiments. (**P*<0.05, ***P*<0.01, ****P*<0.001. NS, not significant)


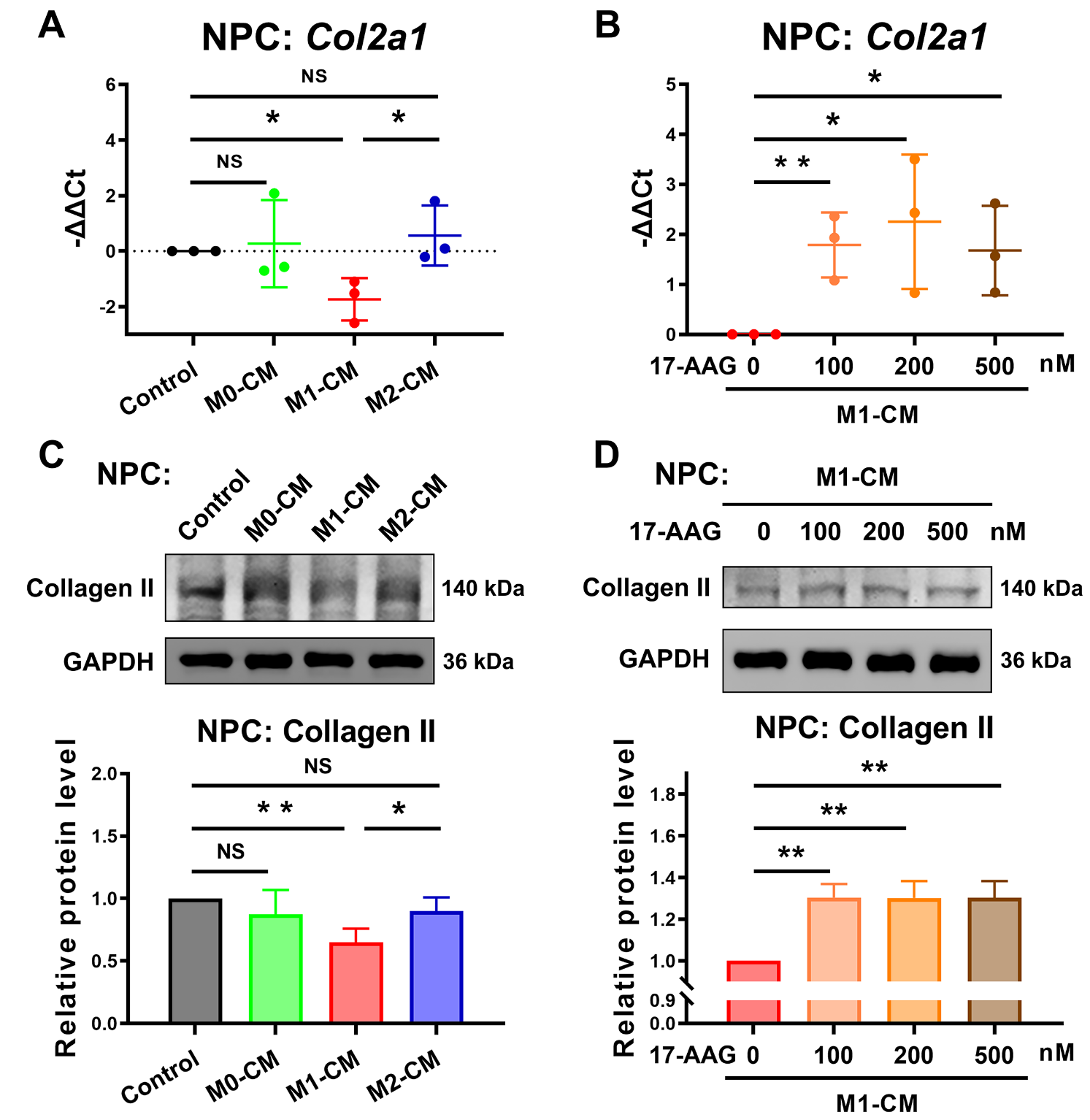


**Supplementary Figure S3. Effects of 17-AAG on Collagen Ⅱ expression in NPCs**

(A, B) The mRNA levels of *Col2a1* in NPCs. (C, D) Representative WB graphs and statistical analysis of Collagen Ⅱ and GAPDH in NPCs. A-D, data were presented as Mean ± SD of three replicates. (**P*<0.05, ***P*<0.01, ****P*<0.001. NS, not significant)


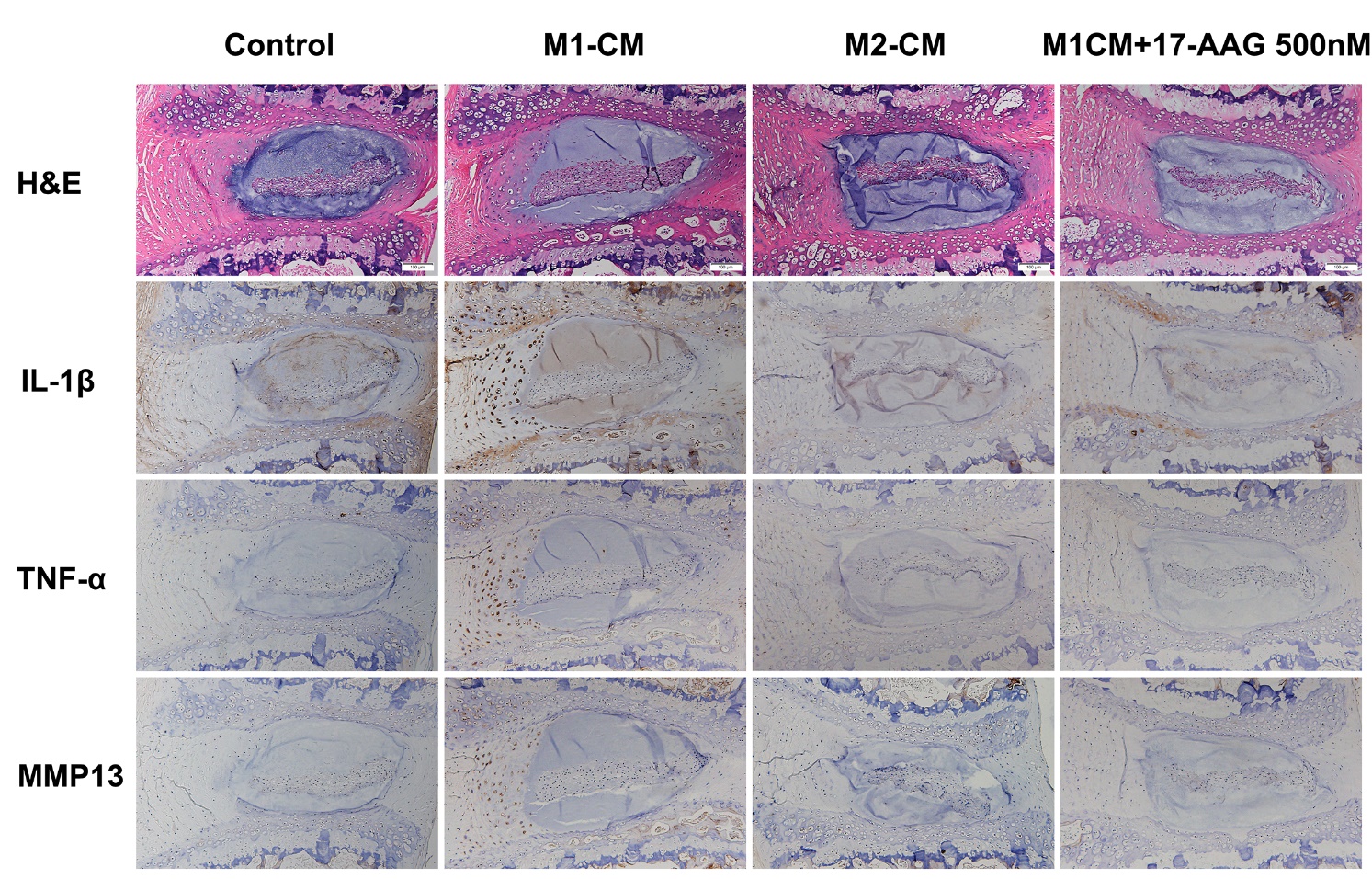


**Supplementary Figure S4. Staining evaluations of *in vitro* cultured IVD tissues**

H&E staining and IHC staining for IL-1β, TNF-α and MMP13 in murine lumbar IVD tissues with the treatment of macrophage CM and 17-AAG (500 nM) *in vitro*.
